# Smokeless tobacco use and its associated factors among secondary school students of Rangeli Municipality of Morang district, Nepal

**DOI:** 10.1371/journal.pone.0313288

**Published:** 2024-11-11

**Authors:** Manish Rajbanshi, Rocky Rajbanshi, Richa Aryal

**Affiliations:** 1 Central Department of Public Health, Institute of Medicine, Tribhuvan University, Kathmandu, Nepal; 2 Padmakanya Multiple Campus, Tribhuvan University, Kathmandu, Nepal; AIIMS Jodhpur: All India Institute of Medical Sciences - Jodhpur, INDIA

## Abstract

**Background:**

The tobacco epidemic is one of the biggest public health threats and the world’s leading cause of preventable death which is responsible for killing 8 million people every year. Adolescents are the vulnerable age group and are at greater risk of any tobacco use including smokeless tobacco (SLT) and nicotine addiction. Tobacco use at a young age increases the risk of various non-communicable diseases (NCDs) such as respiratory illness, asthma, chronic obstructive respiratory disease (COPD), reduced pulmonary function, and cancers. This study aimed to determine the prevalence of smokeless tobacco and its associated factors among secondary school students of grades 11 and 12 in Rangeli Municipality, Nepal.

**Methodology:**

A cross-sectional study was conducted among 355 students from the selected secondary schools. A self-administered questionnaire was used to collect the data. Data was cleaned and then exported to IBM SPSS Statistics 20 for analysis. Participants’ characteristics were described using frequencies, percentages, mean, and standard deviation. Multivariate logistic regression was conducted to determine the association between individual characteristics and prevalence of smokeless tobacco. All the tests were performed at a 95% Confidence Interval (CI) and a p-value less than 0.05.

**Results:**

The mean (±SD) age of the participants was 18.0±1.3 years. Most participants were female (61.1%). The majority of the participants were from grade 11 (58.3%), government schools (60%), and nuclear families (64.8%). This study found that 38.4% and 61.6% of the participants were SLT ever users and never users respectively. Among the ever users, 29.5% were current users and most tried (47.7%) at the age of 10–14 years. Most participants revealed that homes and public places were common sites for SLT use. Paan Masala and Gutkha were the most commonly preferrable SLTs. This study found that age (AOR = 1.5, CI: 1.2–2.2), sex (AOR = 2.6, CI:1.6–4.4), and school type (AOR = 1.8, CI:1.1–3.0) were significantly associated with the prevalence of SLT.

**Conclusion:**

This study found that the prevalence of current SLT users is higher than the national survey. This study concludes that males, young adolescents, and students from private schools are at greater risk of SLT use and are unaware of the consequences on their health. Concerned stakeholders must expand awareness and promote educational programs on the harmful use of tobacco targeting secondary school students. The government organizations, educational institutions, and private organizations jointly work to strengthen the implementation of tobacco cessation programs and tobacco control strategies.

## Introduction

Smokeless tobacco (SLT) is any kind of tobacco that is consumed through mouth or nose without any burning which includes *Khaini/Surti*, *Gutkha*, *Zarda*, *snuff*, and as an ingredient of *Paan masala* [1, 2]. There are approximately 16000 unique flavors available in some markets, many of them appealing to children [3]. Tobacco is one of the biggest epidemic public health threats and kills half of the users who don’t quit in their lifetime [4]. It contributes to poverty by diverting spending on basic needs such as food and shelter to tobacco [5].

Tobacco is one of the world’s leading causes of preventable death. It is responsible for killing nearly 8 million persons every year [5]. The use of smokeless tobacco is highly addictive and damaging to health, containing many cancer-causing toxins. Its use increases the risk of cancers of the head, neck, throat, oesophagus, and oral cavity (including cancer of the mouth, tongue, lip and gums) as well as various dental diseases [6]. An estimated 1.3 billion people use tobacco products globally, among them 80% of users live in low-and middle-income countries (LMICs) [5]. In 2020, 22.3% of the world’s population used tobacco: 36.7% of men and 7.8% of women [5].

Almost 81% of the world’s smokeless tobacco users live in the South-East Asian Region (SEAR) [5]. The proportion of adolescent students who had ever used smokeless tobacco accounted for 11.2% in Egypt, 9% in Kenya, 12.5% in India, 16.2% in Nepal, and 11.9% in Norway [7]. In response to control the tobacco epidemic, all the World Health Organization (WHO) member states adopted the WHO Framework Convention on Tobacco Control (WHO FCTC) in 2003 and currently 182 countries are parties to this convention. Furthermore, the WHO introduced the MPOWER strategy in 2007 which is a practical, and cost-effective initiative to scale up the implementation of the demand reduction provisions of the WHO FCTC [8].

The step-wise approach to non-communicable disease risk factor surveillance (STEPS) survey of 2019 Nepal, reported that 18.3% and 15.3% of the population aged between 15–69 years were current and daily smokeless tobacco users in Nepal respectively [9]. Most current smokeless tobacco users were males (33.3%) compared to females (4.9%) [9]. A national adolescent survey i.e., Global Youth Tobacco Survey 2011 (GYTS) revealed that 16.2% of the individuals aged between 13–15 years were current smokeless tobacco users, among them, boys (19.7%) and girls (12.9%) [10]. These findings highlight a tremendous increase in tobacco users from 7.2% in 2001 to 8% in 2007 to 19.1% in 2011 [10]. In response to control the tobacco epidemic in Nepal, the government of Nepal (GoN) introduced the Tobacco Product Control Act in 2011 ensuring legal provisions to reduce, control, and regulate the import, production, sale, distribution, and consumption of tobacco products [11].

The most prevalent tobacco-related causes of death are cardiovascular disease (53%), chronic respiratory disease (21%), and cancer (8%) [12]. According to the Census 2021 of Nepal, around 20% of the total population was contributed by the young population aged 10–19 years [13]. Adolescents are the most vulnerable age group to any tobacco use and addiction to nicotine. Smoking at a young age increases the risk of various non-communicable diseases such as respiratory illness, asthma, chronic obstructive respiratory disease (COPD), reduced pulmonary function, and cancers [14].

The use of smokeless tobacco is also common, particularly in the Terai belt of Nepal, and is gradually spreading to other parts of Nepal. Previous studies show that tobacco use in Nepal is more common among those with lower education, poor economic status, and adolescents [15]. However, there is limited data on the prevalence, distribution, and trends of tobacco use nationally. Updated information on current usage patterns would aid policymakers in addressing the tobacco epidemic. The prevention of tobacco use among adolescents is the greatest opportunity for preventing non-communicable diseases in Nepal. Hence, this study aimed to determine the prevalence of smokeless tobacco and its associated factors among secondary school students of grades 11 and 12 in Rangeli Municipality, Nepal.

## Methodology

### Study design and settings

A cross-sectional design was conducted among selected secondary schools of Rangeli Municipality. This municipality is located 25 kilometers from Biratnagar and is the headquarters of the Morang district. This municipality includes one government hospital, 2 private hospitals, and 3 health posts [16]. This municipality consists of 19% of the total population who are from the aged group between 10–19 years [13].

### Study population

This study employed those students who were in grades 11 and 12 of the selected secondary school of Rangeli Municipality. Students who were absent and refused to participate during the data collection period were excluded from this study. The students who were unable to hear, or having difficulty in speech and communication were not included in this study.

### Data collection

Data was collected using a self-administered structured questionnaire. Before collecting the data, orientation regarding the study was provided to all the Research Assistants. Principal Investigators and Research Assistants were responsible for collecting the data. No personally identifiable information was collected from study participants or included in the study analysis. Each participant took around 30 minutes to fill out the form.

### Sample size and sampling technique

The sample size was calculated using the Cochran proportionate formula (Z^2^pq/d^2^). The proportion of current tobacco users (p = 29%) among those aged 15–69 years was adopted from the adults survey: STEPS Survey in 2019 [9]. Adjusting 5% margin of error (d), 95% Confidence Interval (CI), and 10% non-response rate, the total sample size for this study was 355 for this study.

The list of all schools in Rangeli Municipality was obtained from the Education Section, Rangeli Municipality. Altogether, there were 8 secondary schools with grades 11 and 12, (2 private and 6 government schools) in the Municipality. The stratified random sampling with a proportionate allocation technique was carried out according to the type of schools. Then, three government and one private school were selected randomly using a lottery method. The average number of students in each school was 110. Then, all the students of grade 11 and grade 12 were recruited in this study from the selected schools. Based on the population proportionate to size, 142 and 213 students were taken from the selected private and government schools respectively in the study.

### Tools and measures

The tool was adapted from the Global Youth Tobacco Survey (GYTS) [17]. The Global Youth Tobacco Survey (GYTS) is an anonymous, self-administered, standardized school-based survey that gathers data on tobacco use among youth to monitor and support tobacco prevention and control programs [18]. The pretesting was done among the 10% of the actual sample size (n = 35) to check the reliability and internal consistency of the study tool. Then, the tool was used to collect the data after obtaining a Cronbach alpha coefficient of 0.82.

The questionnaire was divided into three sections, i.e. the first section included the socio-economic and demographic characteristics of the participants.

The second section included questions regarding smokeless tobacco use. Such as age at first using smokeless tobacco, frequency of tobacco use, days of using tobacco, smoking cessation, source of help-seeking to quit tobacco, and observation of danger signs of tobacco use (yes/no].

The third section included anti-tobacco advertisements by media which contained 8 questions such as age restrictions to buy tobacco products, anti-tobacco campaigns, and anti-tobacco messages in cinemas, magazines, and shops.

### Data management and analysis

The data was systematically entered, edited, filtered, coded, and cross-checked in Epi-Data version 3.1 software. Then, exported to IBM Statistical Package for Social Sciences (SPSS] version 20 for analysis. Frequencies, percentages, mean, and Standard Deviation were calculated to describe the individual characteristics of the participants. Chi-square test and univariate logistic regression were used for the bivariate analysis between SLT ever user and individual characteristics. Then, variables with p-values less than 0.2 in bivariate analysis were included for multivariate regression analysis. Multivariate logistic regression analysis determined the factors associated with smokeless tobacco prevalence. A p-value of less than 0.05 was considered statistically significant in all tests at a 95% Confidence Interval (CI).

### Variable and operational definition

#### Independent variable

It included age (in completed years), sex (male, female, others), grade (11 or 12), type of school (government or private), type of family (nuclear, joint, or extended), ethnicity (Dalit, Janajati, Madhesi, Brahmin/Chettri or Muslim), religion (Hindu, Buddhist, Muslim, or Christian), mother’s and father’s education, and parents’ occupation.

#### Dependent variable

The dependent variable is the prevalence of smokeless tobacco. It was measured in three categories, i.e., current SLT user, never SLT user, and ever SLT user. A current SLT user is defined as one who had used any SLT daily or occasionally during the past 30 days preceding the survey [8]. Never SLT user is defined as one who has never used any SLT in their lifetime [8]. Ever SLT user is defined as one who has used SLT even once in their lifetime [8].

#### Ethical approval

This study was reviewed and approved by the Ethical Review Board (ERB) of the Institute of Medicine, Tribhuvan University [Ref. no: 402(6–11) E2078/079]. A letter of support was obtained from the municipality office and concerned schools. Both written and verbal consent were taken from the participants and their parents/caretakers. The purpose of the study was clearly explained to the participants before data collection. Confidentiality of the information was maintained strictly in the study.

## Results

### Characteristics of the participants

A total of 355 students participated in this study. The mean (±SD) age of the participants was 18.0±1.3 years. The majority of them were female (61.1%), from grade 11 (58.3%), and government school (60%). Most of them belonged to the nuclear family (64.8), Madhesi ethnicity (61.6%), and Hindu religion (95.2%). The majority of the participant’s fathers had completed secondary education (40.3%) while the majority of their mothers did not have any formal education (40.8%) (Table 1).

**Table 1:** Individual characteristics of the study participants.

Characteristics	Frequency (n)	Percentage (%)
**Age (in completed years)**		
Mean ± SD	18.0 ±1.3	
Below 18	144	40.5
18 and above	211	59.5
**Sex**		
Female	217	61.1
Male	138	38.9
**Grade**		
Grade 11	207	58.3
Grade 12	148	41.7
**Type of school**		
Government	213	60.0
Private	142	40.0
**Type of family**		
Nuclear	230	64.8
Joint	105	29.6
Extended	20	5.6
**Ethnicity**		
Madhesi	217	61.1
Janajati	78	22.0
Brahmin/Chettri	32	9.0
Dalit	17	4.8
Muslim	11	3.1
**Religion**		
Hindu	338	95.2
Muslim	13	3.7
Others (Christian, Buddhist)	4	1.1
**Father’s education**		
No formal education	80	22.5
Primary level	107	30.2
Secondary level	143	40.3
Higher secondary level	13	3.7
Bachelor level and above	12	3.3
**Mother’s education**		
No formal education	145	40.8
Primary level	113	31.8
Secondary level	87	24.5
Higher secondary level	7	2.1
Bachelor level and above	3	0.8
**Father’s occupation**		
Agriculture	221	62.3
Businessman	82	23.1
Foreign employment	19	5.4
Labour	14	3.9
Government service	11	3.1
Others (teacher, banker, doctor)	8	2.2
**Mother’s occupation**		
Housemaker	302	85.1
Agriculture	39	11.0
Government service	4	1.1
Others (teacher, business, foreign employment, labour)	10	2.8

### Smokeless tobacco use

Table 2 shows that 38.4% and 61.6% of the participants were SLT ever users and never users, respectively. Among ever user of SLT, 29.5% were current users. Most participants who tried SLT for the first time were at the age of 10 to 14 years (47.7%).

**Table 2:** Prevalence of smokeless tobacco use among the study participants.

Characteristics	Frequency (n)	Percentage (%)
**Have you ever used SLT in your life?**		
Ever used	136	38.4
Never used	219	61.6
**Smokeless tobacco use (n = 136)**		
Current user	42	29.5
Non-current user	94	48.5
**Age when tried SLT for the first time (in years) (n = 136)**		
5–9	13	9.5
10–14	65	47.7
15 and above	58	42.8
**Tobacco use in the past 30 days (n = 136)**		
Within 15 days	95	69.5
More than 16 days	41	30.5
**Frequency of tobacco/smoking use (per day) (n = 136)**		
1–5 times	122	89.5
More than 6 times	14	10.5

### Smokeless tobacco cessation

Table 3 shows that only 19.4% wanted to stop using SLT whereas 24.5% of them tried to stop using it during the past 12 months. Most of the participants (21.4%) didn’t receive help or advice from anyone to stop using SLT.

**Table 3:** Smokeless tobacco cessation among the study participants.

Characteristics	Frequency (n)	Percentage (%)
**Smokeless tobacco cessation (n = 136)**		
**Want to stop using smokeless tobacco now**		
Yes	26	19.4
Not using SLT now	36	25.9
No	74	10.7
**Ever tried to stop using SLT during the past 12 months?**		
Yes	33	24.5
Not using SLT now	29	21.1
No	74	10.4
**Able to stop using SLT if wanted**		
Yes	35	25.6
Not using SLT now	34	24.8
No	67	5.6
**To whom did you seek help or advice to stop using SLT?** *		
Family member	22	16.1
Friend	14	10.1
Program or professional	2	1.6
Programs/professional/friends/family	11	8.4
Did not seek	29	21.4
**Did you see any health warnings on the SLT package in the past 30 days (n = 355)**		
Yes	169	70.1
No	106	29.9
**Did any person working for a tobacco company offer you SLT for free? (n = 355)**		
Yes	16	4.5
No	339	95.5

*Multiple response questions

### Preferred SLT among ever-users tobacco users (n = 136)

**Fig 1:**
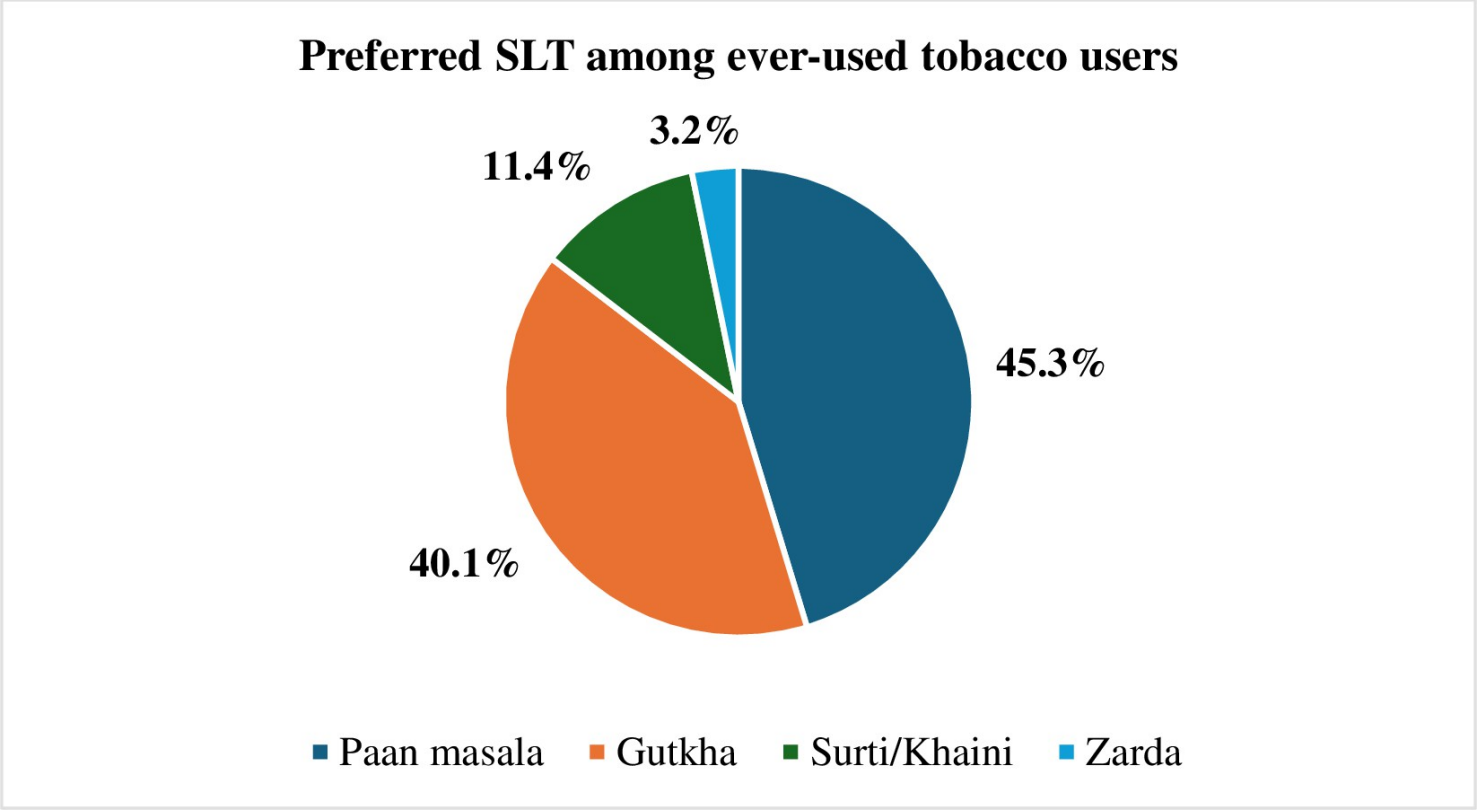
Preferred SLT among ever-used tobacco users.

Fig 1 shows that gutka (40.16%) and paan masala (45.08%) were the most preferred SLT among the participants.

### Opinions on smokeless tobacco use

The majority of participants (63.1%) think that there is no difference in using SLT at celebrations, parties, or other social gatherings. Nearly half of the participants disagreed that people might enjoy using SLT. Home and public places were the most common sites for smoking. Only 42% had heard about the Tobacco Control Law in Nepal (Table 4).

**Table 4:** Opinion on smokeless tobacco use among the study participants.

Characteristics	Frequency (n)	Percentage (%)
**How do you feel when people use SLT at celebrations, parties, or other social gatherings?**		
More comfortable	46	13.0
Less comfortable	85	23.9
No difference	224	63.1
**Agree or disagree “I think people might enjoy using SLT”**		
Strongly agree	8	2.3
Agree	31	8.7
Neutral	44	12.4
Disagree	168	47.3
Strongly disagree	104	29.3
**In your opinion, where do people use SLT the most?**		
Home	129	36.6
Public places	85	23.9
Social events	52	14.6
Schools	45	12.6
Friend’s house	44	12.3
**Have you ever heard of the Tobacco Control Law in Nepal?**		
Yes	149	42.0
No	206	58.0

### The reasons for smoking or chewing tobacco (Multiple response question)

**Fig 2:**
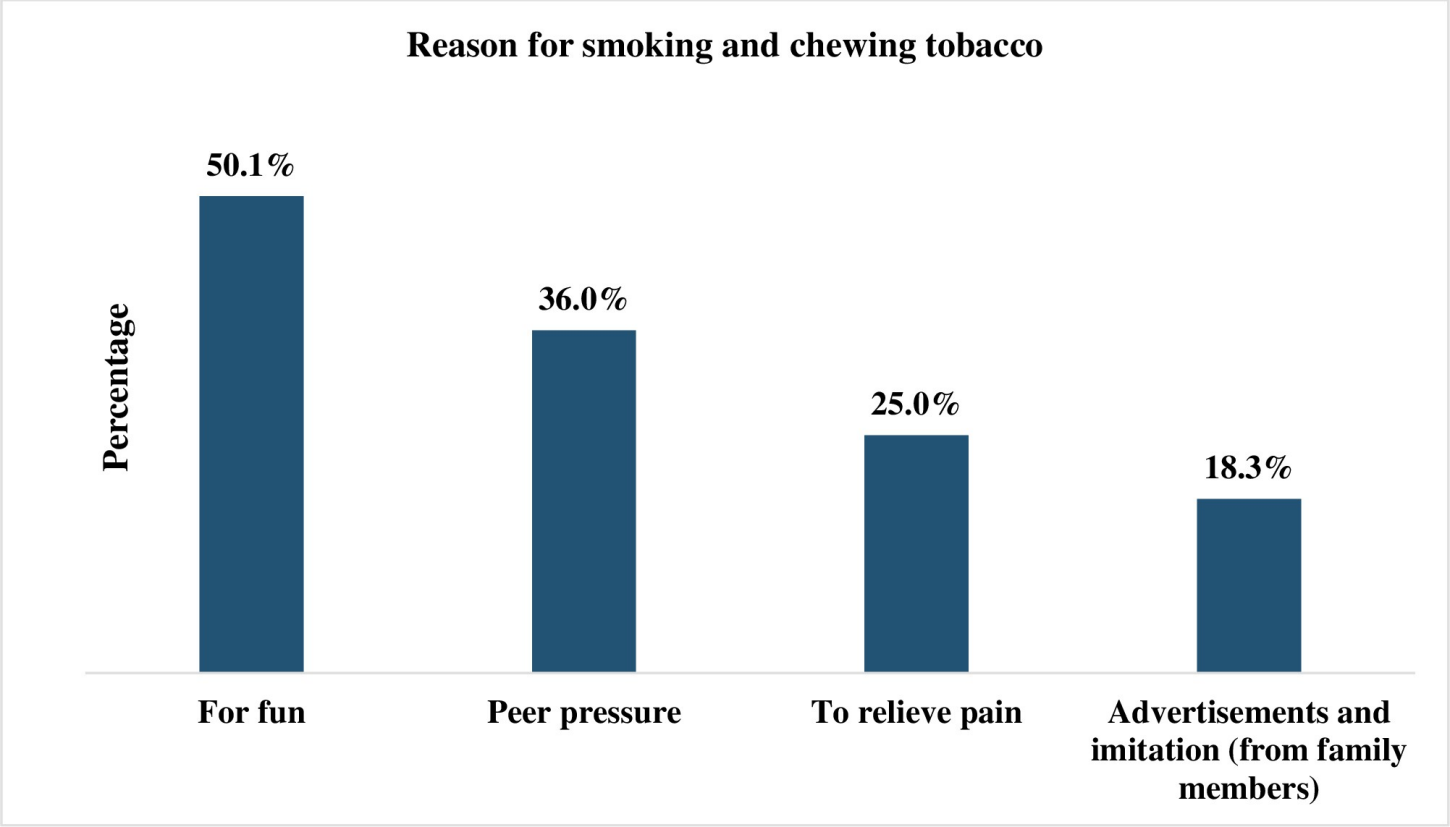
Reasons for smoking and chewing tobacco.

Fig 2 shows that participants revealed that the most common reasons for chewing or smoking were to have fun (50.1%) and peer pressure (26.0%).

### Media anti-tobacco

Most of the participants (85.1%) didn’t ever see any signs stating that adolescents are not allowed to buy tobacco products. The majority of them had seen anti-smoking media messages on television i.e., only a few times (37.5%). While most of the participants were unaware of the anti-tobacco messages in newspapers/magazines (71.3%) and at the point of sale (80.8%) (Table 5).

**Table 5:** Media anti-tobacco among the study participants.

Characteristics	Frequency (n)	Percentage (%)
**Have you ever seen any signs stating that adolescents are not allowed to buy any tobacco products?**		
Yes	53	14.9
No	302	85.1
**Have you ever seen anti-smoking media messages on television?**		
A lot	103	29.0
A few	133	37.5
None	119	33.5
**Have you ever seen anti-smoking messages on hoarding boards?**		
A lot	19	5.4
A few	88	24.8
None	248	69.9
**Have you ever seen anti-smoking media messages in cinema halls?**		
A lot	22	6.2
A few	44	12.4
None	289	81.4
**Have you ever seen anti-smoking media messages in newspapers or magazines?**		
A lot	10	2.8
A few	92	25.9
None	253	71.3
**Have you ever seen anti-smoking media messages at the point of sale (shops, etc.)?**		
A lot	17	4.8
A few	51	14.4
None	287	80.8

### Factors association with the prevalence of smokeless tobacco

Table 6 shows that age (AOR = 1.5, CI: 1.2–2.2), sex (AOR = 2.6, CI:1.6–4.4), and school type (AOR = 1.8, CI:1.1–3.0) and ever seen anti-tobacco messages in newspapers/magazines (AOR = 0.5, CI: 0.5–0.8) were significantly associated with the prevalence of SLT.

**Table 6:** Factors associated with smokeless tobacco use.

Characteristic	Ever used Smokeless tobacco			p-value	Multivariate logistic regression	p-value
	**No**	**Yes**	**COR** **95% CI**		**AOR** **95% CI**	
	**n (%)**	**n (%)**				
	219 (61.6)	136 (38.4)				
**Age**						
Below 18	98 (68.1)	46 (31.9)	Ref	0.042	Ref	0.028
18 and above	121 (57.1)	90 (42.9)	1.6 (1.02–2.4)		1.5 (1.2–2.2)	
**Sex**						
Female	155 (71.4)	62 (28.6)	Ref	0.013	Ref	0.02
Male	64 (46.4)	74 (53.6)	2.8 (1.8–4.5)		2.6 (1.6–4.4)	
**Garde**						
11	131 (63.3)	76 (36.7)	Ref	0.507		
12	88 (59.5)	60 (40.5)	1.1 (0.7–1.8)			
**Type of school**						
Government	144 (67.6)	69 (32.4)	Ref	0.005	Ref	0.011
Private	75 (52.8)	67 (47.2)	1.8 (1.2–2.8		1.8 (1.1–3.0)	
**Type of family**						
Nuclear	81 (64.3)	44 (35.7)	1.2 (0.7–1.8)	0.493		
Joint/Extended	138 (60.0)	92 (40.0)	Ref			
**Ethnicity**						
Janajati	51 (65.4)	26 (34.6)	0.5 (0.2–1.1)	0.124	0.7 (0.3–1.5)	0.154
Madhesi	136 (62.7)	81 (37.3)	0.6 (0.3–1.1)	0.151	0.6 (0.3–1.2)	0.211
Others	32 (52.5)	29 (47.5)	Ref		Ref	
**Father education**						
Informal	49 (61.3)	31 (38.8)	Ref			
Primary	67 (62.6)	40 (37.4)	0.9 (0.5–1.7)	0.849		
Secondary and above	103 (60.9)	65 (39.1)	1.01 (0.5–1.7)	0.963		
**Father occupation**						
Business	46 (56.1)	36 (43.9)	1.4 (0.6–2.8)	0.353		
Agriculture	139 (62.9)	82 (37.1)	1.05 (0.5–1.9)	0.865		
others	34 (64.2)	18 (35.8)	Ref			
**Ever seen anti-tobacco messages about age -restrictions on tobacco sale**						
Yes	27 (50.9)	26 (49.1)	Ref	0.083	Ref	0.310
No	192 (63.6)	110 (36.4)	0.5 (0.3–1.07)		0.7 (0.3–1.3)	
**Ever seen anti-tobacco messages on television**						
Many times	59 (57.3)	4 (42.7)	Ref		Ref	
Few times	75 (56.4)	58 (43.6)	1.03 (0.6–1.7)	0.212	1.0 (0.5–1.9)	0.238
None	85 (71.4)	34 (28.6)	0.5 (0.3–0.7)	0.028	0.5 (0.2–1.1)	0.096
**Ever seen anti-tobacco message in the cinema hall**						
Many times	10 (52.6)	9 (47.4)	Ref			
Few times	54 (61.4)	34 (38.6)	0.7 (0.2–1.8)	0.483		
None	155 (62.5)	93 (37.5)	0.6 (0.2–1.7	0.396		
**Ever seen anti-tobacco messages in newspapers/magazines**						
Many times	10 (45.5)	12 (54.5)	Ref			
Few times	22 (50.0)	22 (50.0)	0.8 (0.2–2.3)	0.728		
None	187 (64.7)	102 (35.3)	0.4 (0.1–1.0)	0.277		
**Ever seen anti-tobacco messages at the point of sale**						
Many times	6 (60.0)	4 (40.0)	Ref			
Few times	57 (62.0)	35 (38.0)	0.9 (0.2–3.4)	0.904		
None	156 (61.7)	97 (38.3)	0.9 (0.2–3.3)	0.916		

## Discussion

The tobacco epidemic is one of the biggest public health threats and the world’s leading cause of preventable death which is responsible for killing 8 million people every year. The use of smokeless tobacco is common, particularly in the Terai belt of Nepal, and is gradually spreading to other parts of Nepal. This study aimed to determine the prevalence of smokeless tobacco and its associated factors among secondary school students of grades 11 and 12 in Rangeli Municipality, Nepal.

This study found that the prevalence of the current smokeless tobacco users was 29.5% among the students. A similar finding was observed in a study conducted in India (24%) [19], the United States (23%) [20] and Nepal (20.1%) [21]. However, our finding is higher than that of the Nepal STEPS Survey 2019 [22]. This study showed that more than one-third tried smokeless tobacco throughout their lifetime which is consistent with a similar study conducted in Kathmandu, Nepal [21]. The noticeable prevalence of SLT is due to low taxation on SLT products [23]. Another obvious reason is that being closer to the Indian border, many imports and illegal production of SLT are growing within the country, making it easily accessible [23].

This study reported that nearly half of the students started using SLT between the ages of 10 to 14 years. This finding is supported by a study done in Bangladesh [24]. Similarly, a study conducted in Kathmandu Valley, Nepal showed that students aged 11 to 19 years started using SLT (62.2%) [25]. In contrast to our study, a previously conducted study in western Nepal revealed that students aged 15 years and above started using SLT [26]. Younger adolescents are more susceptible to tobacco use and smoking due to factors like curiosity, relieving tension, peer pressure, and the influence of media which can lead to starting tobacco use at a younger age [25, 26].

A maximum proportion of secondary school students used tobacco or smoked at most 5 times per day (89.5%) which is higher than the annual survey conducted by the Department of Health Services, Nepal in 2023/24 [27].

In this study, one in five current SLT users want to stop using tobacco which is similar to a study conducted among adolescents in Bangladesh [24]. Furthermore, one in four (24.5%) current SLT users ever tried to stop using SLT which is higher than that of the Nepal STEP Survey 2019 among adults (17.9%) [22]. The school students might have seen or heard about the burden of tobacco leading to various diseases like cardiovascular diseases, diabetes and kidney diseases, neoplasms, and non-communicable diseases like tuberculosis, and cancer [28]. However, this finding is lower than the students who tried to quit the habit of using tobacco in western Nepal [26] and the study conducted on public health undergraduates in Nepal [25].

Of those participants who received help or advice to stop using SLT, 16.1% sought it from family members whereas the Nepal STEPS Survey 2019 reported that 21% of SLT users sought help from healthcare providers [22]. Contradictory, this finding is lower than a similar study done in Kathmandu Valley, Nepal which showed that 73.8% ever users received help or advice to stop using tobacco and cigarettes [25]. However, 21.4% of the ever SLT users did not seek help from anyone in this study. It might be because adolescents often believe smokeless tobacco is a safer alternative to smoking, making them less likely to seek help for quitting tobacco use [29].

Unfortunately, nearly one-third of the participants had not seen health warnings on SLT packages, which is consistent with a study conducted in India (27%) [30]. The trend in the decrease in noticing the health warning labels among SLT quitters could be explained by the fact that they were no longer regularly exposed to SLT packaging [30]. Another reason is, a substantial minority were not even aware that there were health warning labels on SLT products [30]. Most SLT users have seen the warning signs on tobacco packages, but those users who cannot read, have not seen the warning messages on tobacco packages [31].

Most of the students preferred Paan masala (45.08%) and Gutkha (40.16%) which is slightly comparable with the study conducted in western Nepal i.e., Paan masala (42.9%) and Gutkha (20.7%) [26] and another study conducted on Nepalese students [32]. However, Zarda was preferred the most in Bangladesh [24].

According to our study, most students revealed that home and public places were the most preferable sites for tobacco use [31]. This finding is supported by a previously conducted study in Nepal among Nepalese students [32] and Sri Lanka among males [31]. It is due to tobacco users feel safe at home as they might have more freedom [31, 32]. This demonstrates that they are somewhat in control of their smoking habits and public place behaviors [26, 31, 32].

To have fun, peer pressure, to relieve pain and the influence of family members/movie actors were the influencing factors for tobacco consumption. It is comparable to a study conducted in Nepal [26, 32] and systematic review done to investigate the reasons for using smokeless tobacco [33]. It is due to SLT products are mostly imported from India and are easily accessible to the public [33]. Another reason is, that the general public is often unaware of the health risks linked to smokeless tobacco use [32], misconceptions about the health benefits of these products, perpetuated by traditional health messages and long-held beliefs, may encourage their consumption [30, 32, 33]. The primary factor for initiation was the influence of family members. SLT users also believed that it protected them from evil spirits and dangerous reptiles [33]. Also, a significant proportion of younger adolescents are still unaware of the Tobacco Control Law within the country of Nepal [25].

Only a minority of the participants had seen anti-smoking media messages quite a lot on television, and even fewer in cinema halls. On the contrary, this finding is lower compared to the Nepal STEPS Survey 2019 among adults [22]. This finding suggested that anti-tobacco media messages should be promoted and expanded to the community level, workplaces, and educational institutions to aware of the harmful use of tobacco and to control tobacco consumption [31, 34].

The higher age group was 1.5 times more likely to use SLT than the lower age group in this study. A similar pattern was observed in a study conducted by Pérez et al., 2022 [35], in Kathmandu, Nepal [21] and among public health undergraduates in Nepal [25]. These are emerging adults who have moved away from their families for the first time and are faced with unprecedented freedom and might have received higher pocket money [35]. These factors could have made tobacco products more accessible to them. Young adults may be more likely to be around others who use tobacco, like friends or family members [25, 33]. This social influence can make them more likely to experiment and increase their impulse, making them more susceptible to peer pressure.

Male students had 2.6 times more odds of using smokeless tobacco than females. It is supported by several studies done in Sudan [7], India [19], Bangladesh [24], South-East Asia [36] and Nepal [23]. In the Nepalese context, smoking and other forms of tobacco are considered as acceptable behavior for men but not for women [23]. Male students are easily influenced by peer pressure for the initiation of tobacco use [35, 37]. Peer influence, rebelliousness, and thrill-seeking appear to predict smokeless tobacco initiation strongly among male youth [33]. Additionally, social norms and the prohibition of tobacco use can be one of the factors responsible for the lower prevalence of tobacco use in the female population in Southeast Asian countries [28].

Tobacco consumption was higher among private school students (24.5%) than government school students (19.6%). This finding is coherent with the study conducted among Nepalese students [32] and a similar study done in Cameroon [38]. This study demonstrated that private school students had 1.8 times higher odds of using SLT than government school students. In contrast, a study conducted in India revealed no difference in SLT consumption between private and government colleges [37]. This might be because private schools might receive more media attention regarding student behavior, making any isolated incident of tobacco use seem more prevalent.

This study depicted that family type was not associated with the prevalence of SLT which is in disagreement with the study conducted among Nepalese students [32] and Sudanese adolescents [7]. Similarly, the education of parents and their occupations were not associated with SLT consumption among the students [19, 39, 40]. However, several studies reported that family history of using tobacco/smoking, family’s monthly income, pocket money, and tobacco control policies are the determinants of tobacco consumption among school students.

### Strengths and limitations

This study provides additional evidence to limited studies conducted on smokeless tobacco among school students. This study provides supplementary information for further studies such as opinions of students on SLT, and media anti-tobacco that could be used as evidence for school-based intervention design.

Information on smokeless tobacco use is based on self-reported, so there might be a chance of response and recall bias that might influence the results. Since our study is based on adolescents in schools, these findings might have limited generalizability to adolescents of other age groups and those out of school. Furthermore, this study is based on a cross-sectional design, no causal relationship can be established between smokeless tobacco and its associated factors.

## Conclusion

This study demonstrated that young students, males, and students from private schools are at a greater risk of SLT use. Meanwhile, most students believed that the reasons for SLT use are to have fun, due to peer pressure, and to relieve pain without knowing the consequences on their health. Still a notable number of students are unaware of anti-tobacco messages on tobacco products, cinema halls, and at the point of sale.

Hence, concerned stakeholders must expand awareness and promote educational programs on the harmful use of tobacco targeting school students. All level governments, educational institutions, and private organizations should jointly work to strengthen the implementation of tobacco cessation programs and tobacco control strategies. Additionally, school-based interventions and programs should be carried out to control and prevent all kinds of tobacco use especially targeting school-going adolescents, males, and private schools.

## Supporting information

S1 Dataset Data for analysis. (CSV)

## Abbreviations

AOR: Adjusted Odd Ratio
CI: Confidence Interval
COPD: Chronic Obstructive Respiratory Disease
DoHS: Department of Health Service
FCTC: Framework Convention on Tobacco Control
GoN: Government of Nepal
GYTS: Global Youth Tobacco Survey
NDHS: Nepal Demographic Health Survey
RM: Rangeli Municipality
SD: Standard Deviation
SLT: Smokeless Tobacco
STEPS: Step-wise approach to non-communicable disease risk factor Surveillance
WHO: World Health Organization
